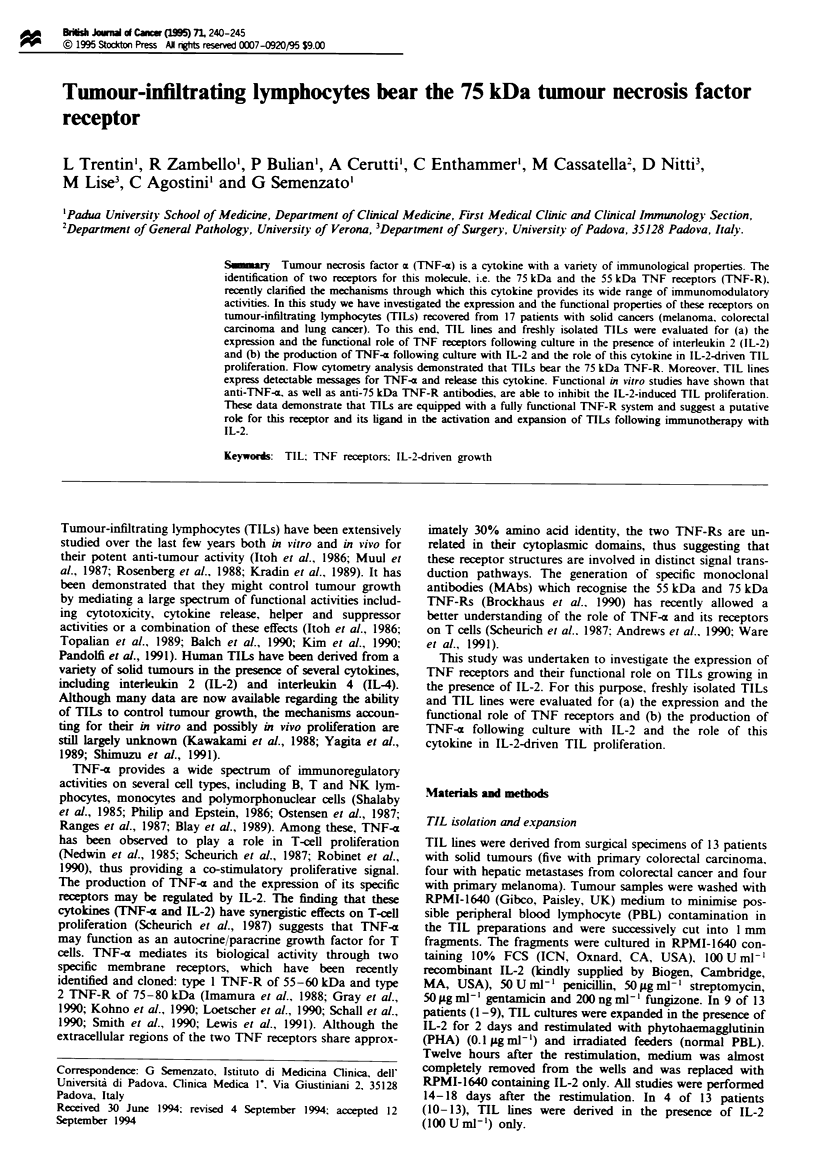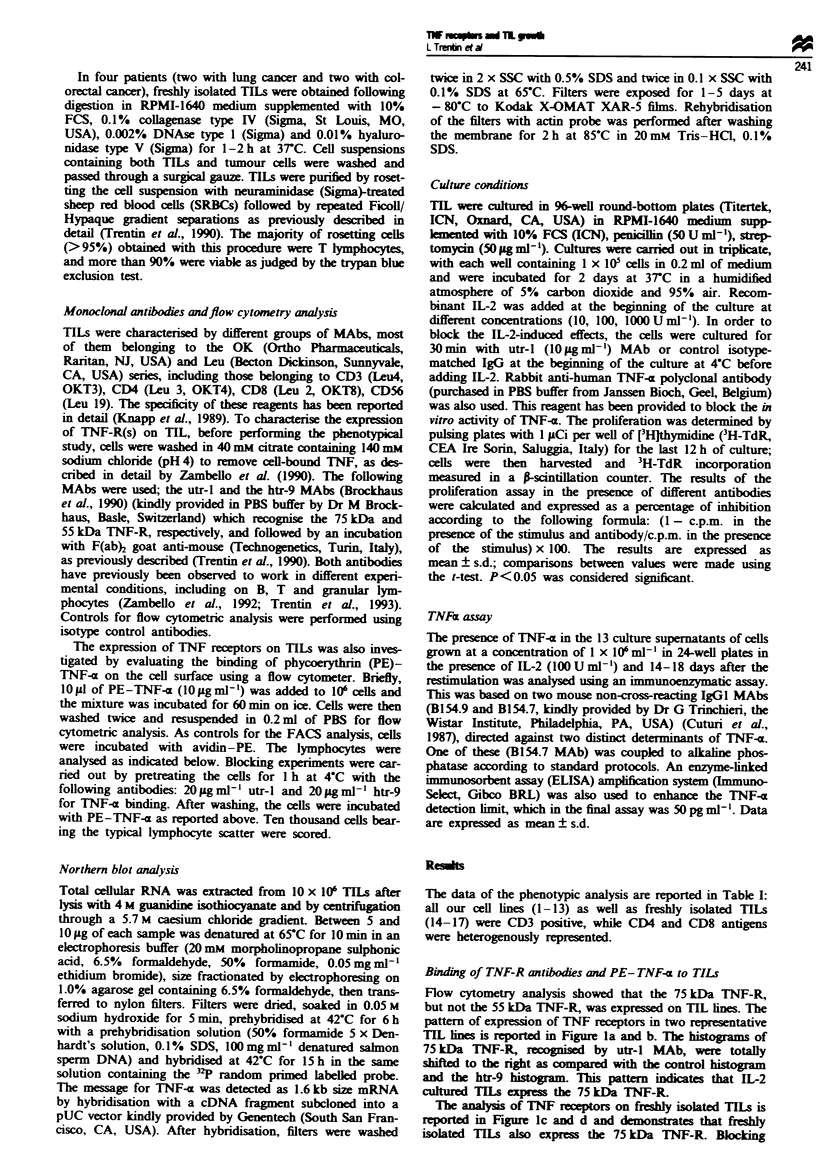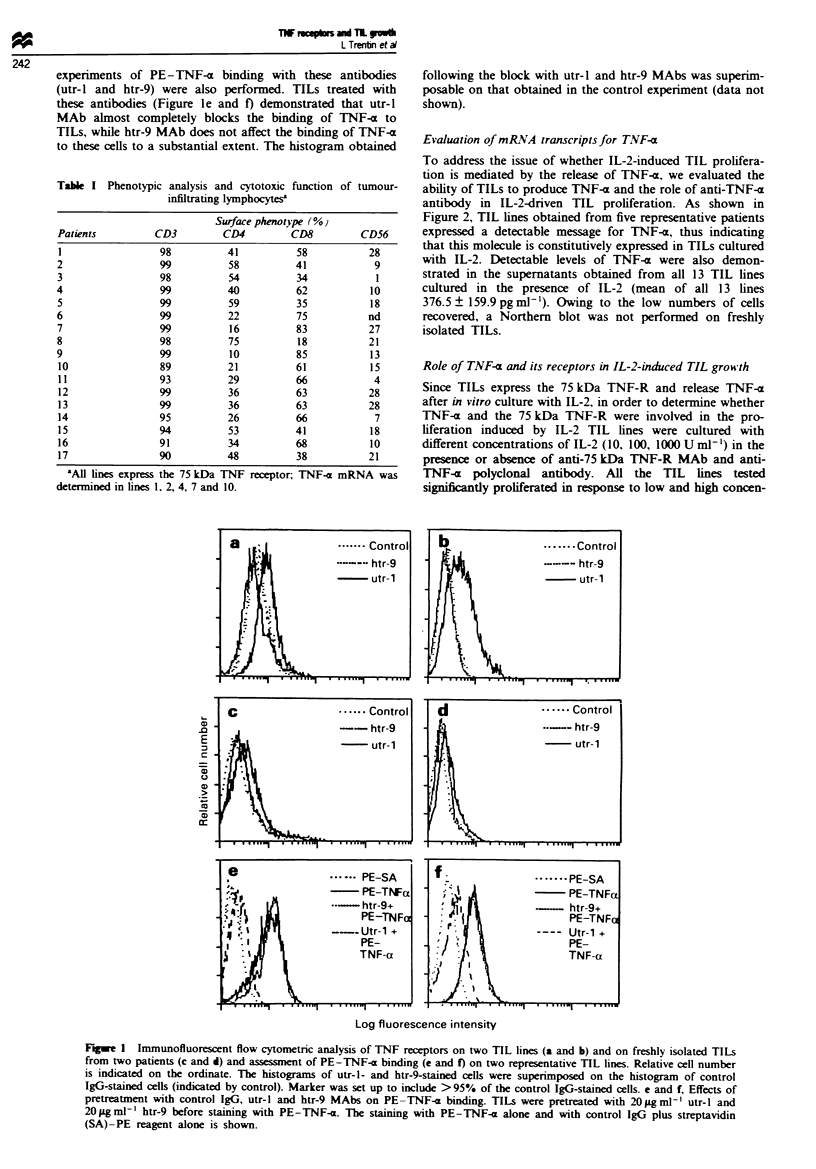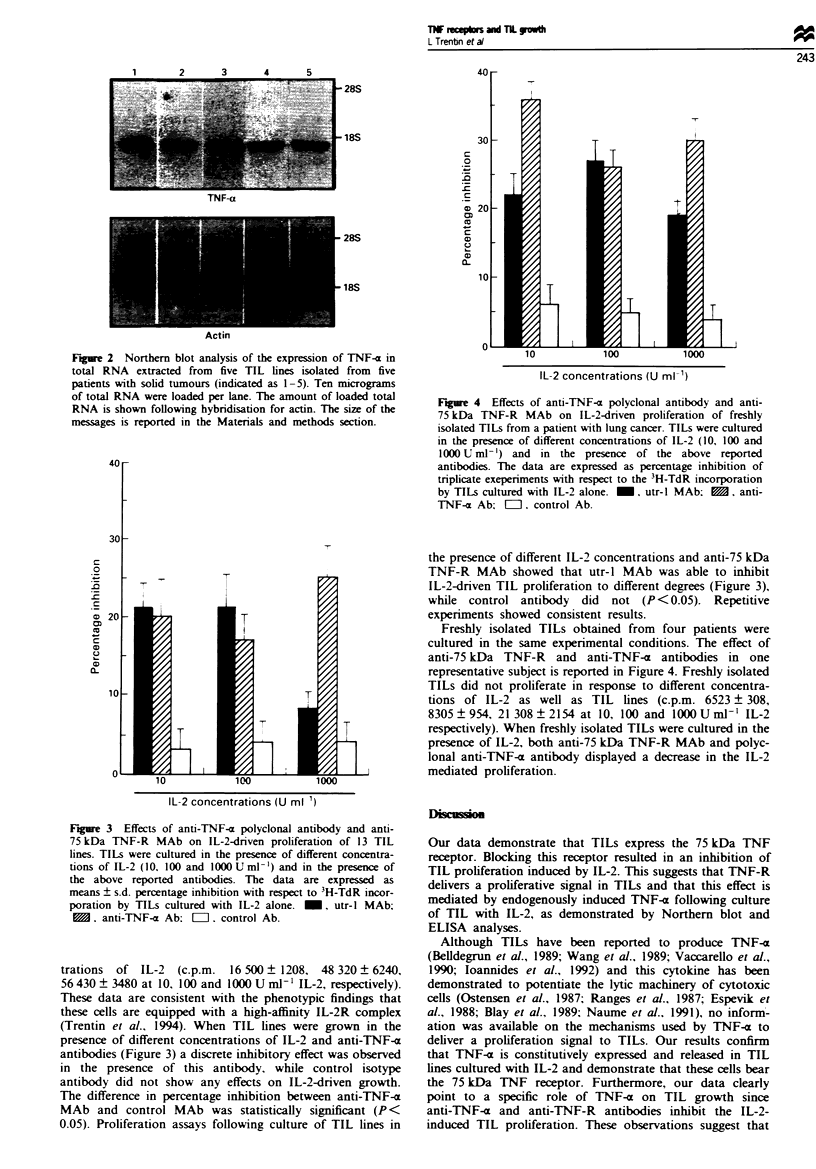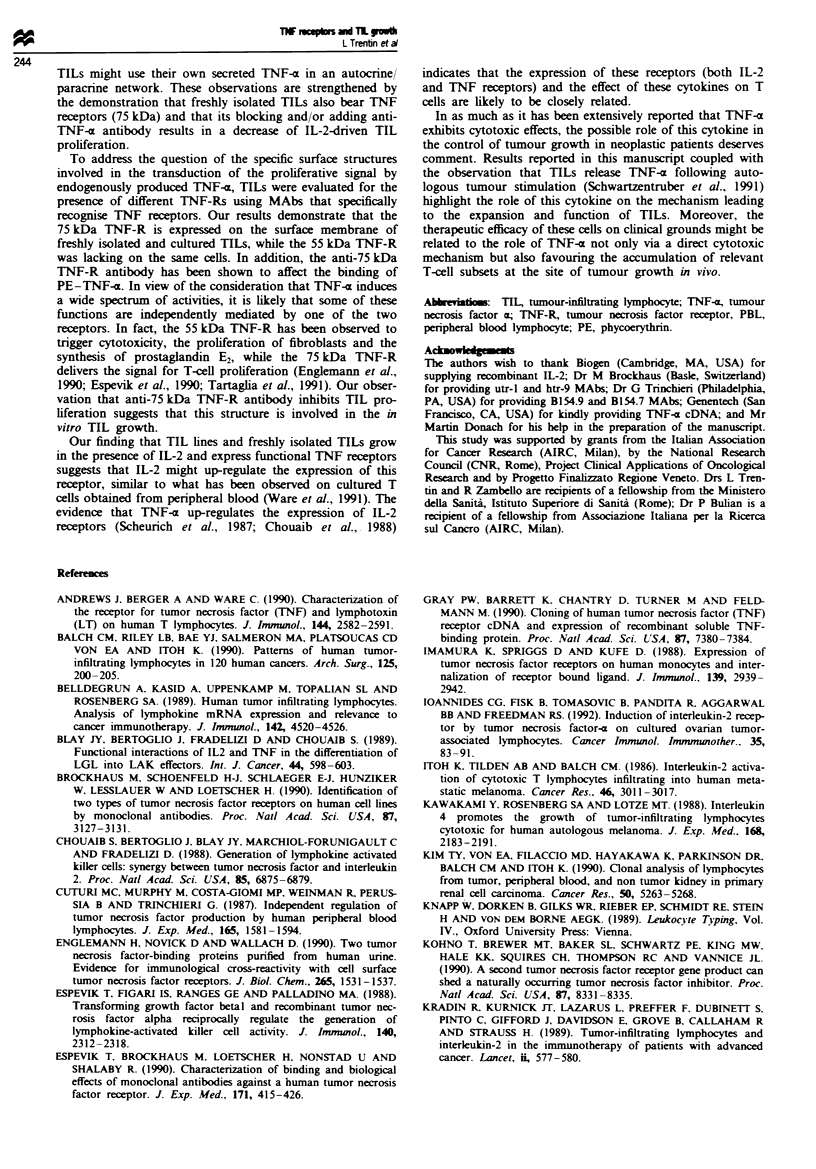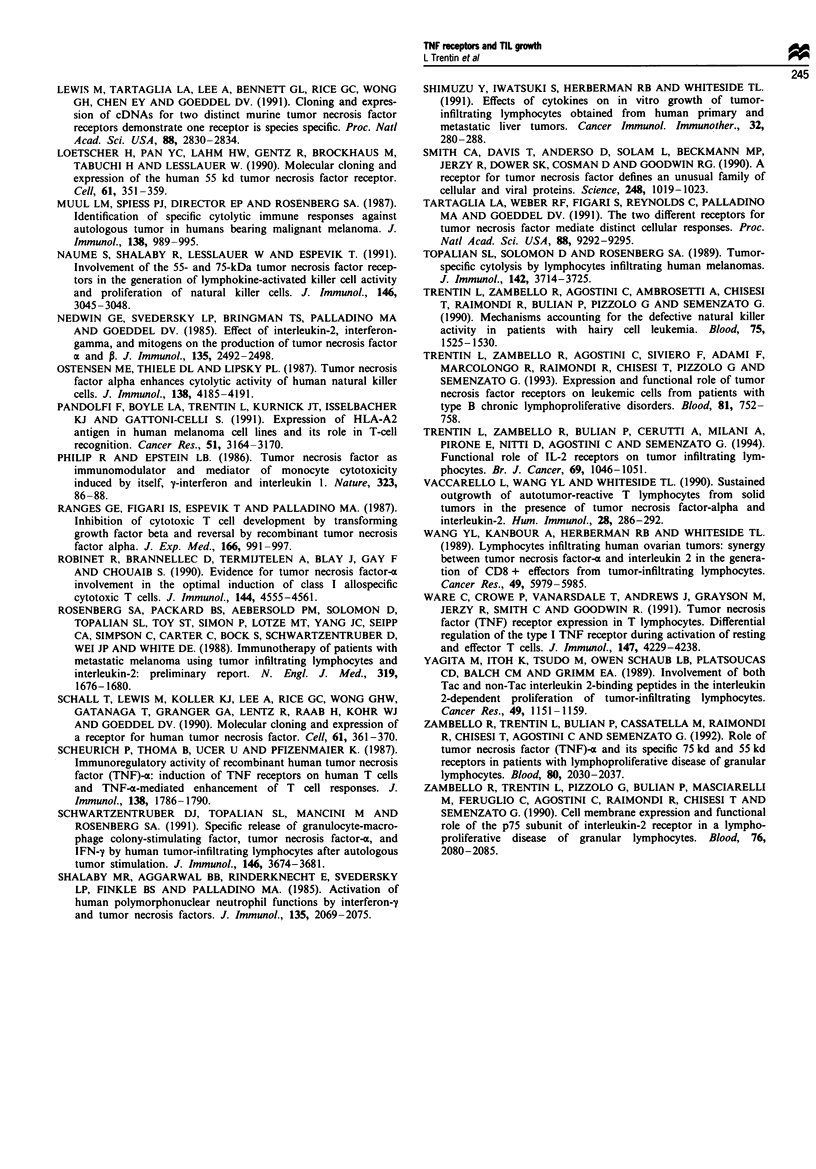# Tumour-infiltrating lymphocytes bear the 75 kDa tumour necrosis factor receptor.

**DOI:** 10.1038/bjc.1995.50

**Published:** 1995-02

**Authors:** L. Trentin, R. Zambello, P. Bulian, A. Cerutti, C. Enthammer, M. Cassatella, D. Nitti, M. Lise, C. Agostini, G. Semenzato

**Affiliations:** Padua University School of Medicine, Department of Clinical Medicine, Italy.

## Abstract

**Images:**


British Journal of Cancer (1995) 71, 240-245

© 1995 Stockton Press All rights reserved 0007-0920/95 $9.00

### Tumour-infiltrating lymphocytes bear the 75 kDa tumour necrosis factor receptor

L Trentin[1], R Zambello[1], P Bulian[1], A Cerutti[1], C Enthammer[1], M Cassatella[2], D Nitti[3], M Lise[3], C Agostini[1] and G Semenzato[1]

[1]*Padua University School of Medicine, Department of Clinical Medicine, First Medical Clinic and Clinical Immunology Section,* [2]*Department of General Pathology, University of Verona,* [3]*Department of Surgery, University of Padova, 35128 Padova, Italy.*

**Summary** Tumour necrosis factor α (TNF-α) is a cytokine with a variety of immunological properties. The identification of two receptors for this molecule, i.e. the 75 kDa and the 55 kDa TNF receptors (TNF-R), recently clarified the mechanisms through which this cytokine provides its wide range of immunomodulatory activities. In this study we have investigated the expression and the functional properties of these receptors on tumour-infiltrating lymphocytes (TILs) recovered from 17 patients with solid cancers (melanoma, colorectal carcinoma and lung cancer). To this end, TIL lines and freshly isolated TILs were evaluated for (a) the expression and the functional role of TNF receptors following culture in the presence of interleukin 2 (IL-2) and (b) the production of TNF-α following culture with IL-2 and the role of this cytokine in IL-2-driven TIL proliferation. Flow cytometry analysis demonstrated that TILs bear the 75 kDa TNF-R. Moreover, TIL lines express detectable messages for TNF-α and release this cytokine. Functional *in vitro* studies have shown that anti-TNF-α, as well as anti-75 kDa TNF-R antibodies, are able to inhibit the IL-2-induced TIL proliferation. These data demonstrate that TILs are equipped with a fully functional TNF-R system and suggest a putative role for this receptor and its ligand in the activation and expansion of TILs following immunotherapy with IL-2.

**Keywords**: TIL; TNF receptors; IL-2-driven growth

Tumour-infiltrating lymphocytes (TILs) have been extensively studied over the last few years both *in vitro* and *in vivo* for their potent anti-tumour activity (Itoh *et al*., 1986; Muul *et al*., 1987; Rosenberg *et al*., 1988; Kradin *et al*., 1989). It has been demonstrated that they might control tumour growth by mediating a large spectrum of functional activities including cytotoxicity, cytokine release, helper and suppressor activities or a combination of these effects (Itoh *et al*., 1986; Topalian *et al*., 1989; Balch *et al*., 1990; Kim *et al*., 1990; Pandolfi *et al*., 1991). Human TILs have been derived from a variety of solid tumours in the presence of several cytokines, including interleukin 2 (IL-2) and interleukin 4 (IL-4). Although many data are now available regarding the ability of TILs to control tumour growth, the mechanisms accounting for their *in vitro* and possibly *in vivo* proliferation are still largely unknown (Kawakami *et al*., 1988; Yagita *et al*., 1989; Shimuzu *et al*., 1991).

TNF-α provides a wide spectrum of immunoregulatory activities on several cell types, including B, T and NK lymphocytes, monocytes and polymorphonuclear cells (Shalaby *et al*., 1985; Philip and Epstein, 1986; Ostensen *et al*., 1987; Ranges *et al*., 1987; Blay *et al*., 1989). Among these, TNF-α has been observed to play a role in T-cell proliferation (Nedwin *et al*., 1985; Scheurich *et al*., 1987; Robinet *et al*., 1990), thus providing a co-stimulatory proliferative signal. The production of TNF-α and the expression of its specific receptors may be regulated by IL-2. The finding that these cytokines (TNF-α and IL-2) have synergistic effects on T-cell proliferation (Scheurich *et al*., 1987) suggests that TNF-α may function as an autocrine paracrine growth factor for T cells. TNF-α mediates its biological activity through two specific membrane receptors, which have been recently identified and cloned: type 1 TNF-R of 55-60 kDa and type 2 TNF-R of 75-80 kDa (Imamura *et al*., 1988; Gray *et al*., 1990; Kohno *et al*., 1990; Loetscher *et al*., 1990; Schall *et al*., 1990; Smith *et al*., 1990; Lewis *et al*., 1991). Although the extracellular regions of the two TNF receptors share approximately 30% amino acid identity, the two TNF-Rs are unrelated in their cytoplasmic domains, thus suggesting that these receptor structures are involved in distinct signal transduction pathways. The generation of specific monoclonal antibodies (MAbs) which recognise the 55 kDa and 75 kDa TNF-Rs (Brockhaus *et al*., 1990) has recently allowed a better understanding of the role of TNF-α and its receptors on T cells (Scheurich *et al*., 1987; Andrews *et al*., 1990; Ware *et al*., 1991).

This study was undertaken to investigate the expression of TNF receptors and their functional role on TILs growing in the presence of IL-2. For this purpose, freshly isolated TILs and TIL lines were evaluated for (a) the expression and the functional role of TNF receptors and (b) the production of TNF-α following culture with IL-2 and the role of this cytokine in IL-2-driven TIL proliferation.

### Materials and methods

### *TIL isolation and expansion*

TIL lines were derived from surgical specimens of 13 patients with solid tumours (five with primary colorectal carcinoma, four with hepatic metastases from colorectal cancer and four with primary melanoma). Tumour samples were washed with RPMI-1640 (Gibco, Paisley, UK) medium to minimise possible peripheral blood lymphocyte (PBL) contamination in the TIL preparations and were successively cut into 1 mm fragments. The fragments were cultured in RPMI-1640 containing 10% FCS (ICN, Oxnard, CA, USA), 100 U $ml^{-1}$ recombinant IL-2 (kindly supplied by Biogen, Cambridge, MA, USA), 50 U $ml^{-1}$ penicillin, 50 $\mu g\ ml^{-1}$ streptomycin, 50 $\mu g\ ml^{-1}$ gentamicin and 200 ng $ml^{-1}$ fungizone. In 9 of 13 patients (1-9), TIL cultures were expanded in the presence of IL-2 for 2 days and restimulated with phytohaemagglutinin (PHA) (0.1 $\mu g\ ml^{-1}$) and irradiated feeders (normal PBL). Twelve hours after the restimulation, medium was almost completely removed from the wells and was replaced with RPMI-1640 containing IL-2 only. All studies were performed 14-18 days after the restimulation. In 4 of 13 patients (10-13), TIL lines were derived in the presence of IL-2 (100 U $ml^{-1}$) only.

Correspondence: G Semenzato, Istituto di Medicina Clinica, dell' Università di Padova, Clinica Medica 1°, Via Giustiniani 2, 35128 Padova, Italy
Received 30 June 1994; revised 4 September 1994; accepted 12 September 1994

In four patients (two with lung cancer and two with colorectal cancer), freshly isolated TILs were obtained following digestion in RPMI-1640 medium supplemented with 10% FCS, 0.1% collagenase type IV (Sigma, St Louis, MO, USA), 0.002% DNAse type 1 (Sigma) and 0.01% hyaluronidase type V (Sigma) for 1–2 h at 37°C. Cell suspensions containing both TILs and tumour cells were washed and passed through a surgical gauze. TILs were purified by rosetting the cell suspension with neuraminidase (Sigma)-treated sheep red blood cells (SRBCs) followed by repeated Ficoll/Hypaque gradient separations as previously described in detail (Trentin *et al.*, 1990). The majority of rosetting cells (>95%) obtained with this procedure were T lymphocytes, and more than 90% were viable as judged by the trypan blue exclusion test.

### *Monoclonal antibodies and flow cytometry analysis*

TILs were characterised by different groups of MAbs, most of them belonging to the OK (Ortho Pharmaceuticals, Raritan, NJ, USA) and Leu (Becton Dickinson, Sunnyvale, CA, USA) series, including those belonging to CD3 (Leu4, OKT3), CD4 (Leu 3, OKT4), CD8 (Leu 2, OKT8), CD56 (Leu 19). The specificity of these reagents has been reported in detail (Knapp *et al.*, 1989). To characterise the expression of TNF-R(s) on TIL, before performing the phenotypical study, cells were washed in 40 mM citrate containing 140 mM sodium chloride (pH 4) to remove cell-bound TNF, as described in detail by Zambello *et al.* (1990). The following MAbs were used: the utr-1 and the htr-9 MAbs (Brockhaus *et al.*, 1990) (kindly provided in PBS buffer by Dr M Brockhaus, Basle, Switzerland) which recognise the 75 kDa and 55 kDa TNF-R, respectively, and followed by an incubation with $F(ab)_2$ goat anti-mouse (Technogenetics, Turin, Italy), as previously described (Trentin *et al.*, 1990). Both antibodies have previously been observed to work in different experimental conditions, including on B, T and granular lymphocytes (Zambello *et al.*, 1992; Trentin *et al.*, 1993). Controls for flow cytometric analysis were performed using isotype control antibodies.

The expression of TNF receptors on TILs was also investigated by evaluating the binding of phycoerythrin (PE)–TNF-α on the cell surface using a flow cytometer. Briefly, 10 µl of PE–TNF-α (10 µg $ml^{-1}$) was added to $10^6$ cells and the mixture was incubated for 60 min on ice. Cells were then washed twice and resuspended in 0.2 ml of PBS for flow cytometric analysis. As controls for the FACS analysis, cells were incubated with avidin–PE. The lymphocytes were analysed as indicated below. Blocking experiments were carried out by pretreating the cells for 1 h at 4°C with the following antibodies: 20 µg $ml^{-1}$ utr-1 and 20 µg $ml^{-1}$ htr-9 for TNF-α binding. After washing, the cells were incubated with PE–TNF-α as reported above. Ten thousand cells bearing the typical lymphocyte scatter were scored.

### *Northern blot analysis*

Total cellular RNA was extracted from 10 × $10^6$ TILs after lysis with 4 M guanidine isothiocyanate and by centrifugation through a 5.7 M caesium chloride gradient. Between 5 and 10 µg of each sample was denatured at 65°C for 10 min in an electrophoresis buffer (20 mM morpholinopropane sulphonic acid, 6.5% formaldehyde, 50% formamide, 0.05 mg $ml^{-1}$ ethidium bromide), size fractionated by electrophoresing on 1.0% agarose gel containing 6.5% formaldehyde, then transferred to nylon filters. Filters were dried, soaked in 0.05 M sodium hydroxide for 5 min, prehybridised at 42°C for 6 h with a prehybridisation solution (50% formamide 5 × Denhardt's solution, 0.1% SDS, 100 mg $ml^{-1}$ denatured salmon sperm DNA) and hybridised at 42°C for 15 h in the same solution containing the $^{32}P$ random primed labelled probe. The message for TNF-α was detected as 1.6 kb size mRNA by hybridisation with a cDNA fragment subcloned into a pUC vector kindly provided by Genentech (South San Francisco, CA, USA). After hybridisation, filters were washed twice in 2 × SSC with 0.5% SDS and twice in 0.1 × SSC with 0.1% SDS at 65°C. Filters were exposed for 1–5 days at – 80°C to Kodak X-OMAT XAR-5 films. Rehybridisation of the filters with actin probe was performed after washing the membrane for 2 h at 85°C in 20 mM Tris–HCl, 0.1% SDS.

### *Culture conditions*

TIL were cultured in 96-well round-bottom plates (Titertek, ICN, Oxnard, CA, USA) in RPMI-1640 medium supplemented with 10% FCS (ICN), penicillin (50 U $ml^{-1}$), streptomycin (50 µg $ml^{-1}$). Cultures were carried out in triplicate, with each well containing 1 × $10^5$ cells in 0.2 ml of medium and were incubated for 2 days at 37°C in a humidified atmosphere of 5% carbon dioxide and 95% air. Recombinant IL-2 was added at the beginning of the culture at different concentrations (10, 100, 1000 U $ml^{-1}$). In order to block the IL-2-induced effects, the cells were cultured for 30 min with utr-1 (10 µg $ml^{-1}$) MAb or control isotype-matched IgG at the beginning of the culture at 4°C before adding IL-2. Rabbit anti-human TNF-α polyclonal antibody (purchased in PBS buffer from Janssen Bioch, Geel, Belgium) was also used. This reagent has been provided to block the *in vitro* activity of TNF-α. The proliferation was determined by pulsing plates with 1 µCi per well of [$^3$H]thymidine ($^3$H-TdR, CEA Ire Sorin, Saluggia, Italy) for the last 12 h of culture; cells were then harvested and $^3$H-TdR incorporation measured in a β-scintillation counter. The results of the proliferation assay in the presence of different antibodies were calculated and expressed as a percentage of inhibition according to the following formula: (1 – c.p.m. in the presence of the stimulus and antibody/c.p.m. in the presence of the stimulus) × 100. The results are expressed as mean ± s.d.; comparisons between values were made using the *t*-test. $P<0.05$ was considered significant.

### *TNFα assay*

The presence of TNF-α in the 13 culture supernatants of cells grown at a concentration of 1 × $10^6$ $ml^{-1}$ in 24-well plates in the presence of IL-2 (100 U $ml^{-1}$) and 14–18 days after the restimulation was analysed using an immunoenzymatic assay. This was based on two mouse non-cross-reacting IgG1 MAbs (B154.9 and B154.7, kindly provided by Dr G Trinchieri, the Wistar Institute, Philadelphia, PA, USA) (Cuturi *et al.*, 1987), directed against two distinct determinants of TNF-α. One of these (B154.7 MAb) was coupled to alkaline phosphatase according to standard protocols. An enzyme-linked immunosorbent assay (ELISA) amplification system (Immuno-Select, Gibco BRL) was also used to enhance the TNF-α detection limit, which in the final assay was 50 pg $ml^{-1}$. Data are expressed as mean ± s.d.

### Results

The data of the phenotypic analysis are reported in Table I: all our cell lines (1–13) as well as freshly isolated TILs (14–17) were CD3 positive, while CD4 and CD8 antigens were heterogenously represented.

### *Binding of TNF-R antibodies and PE–TNF-α to TILs*

Flow cytometry analysis showed that the 75 kDa TNF-R, but not the 55 kDa TNF-R, was expressed on TIL lines. The pattern of expression of TNF receptors in two representative TIL lines is reported in Figure 1a and b. The histograms of 75 kDa TNF-R, recognised by utr-1 MAb, were totally shifted to the right as compared with the control histogram and the htr-9 histogram. This pattern indicates that IL-2 cultured TILs express the 75 kDa TNF-R.

The analysis of TNF receptors on freshly isolated TILs is reported in Figure 1c and d and demonstrates that freshly isolated TILs also express the 75 kDa TNF-R. Blocking experiments of PE–TNF-α binding with these antibodies (utr-1 and htr-9) were also performed. TILs treated with these antibodies (Figure 1e and f) demonstrated that utr-1 MAb almost completely blocks the binding of TNF-α to TILs, while htr-9 MAb does not affect the binding of TNF-α to these cells to a substantial extent. The histogram obtained following the block with utr-1 and htr-9 MAbs was superimposable on that obtained in the control experiment (data not shown).

**Table I** Phenotypic analysis and cytotoxic function of tumour-infiltrating lymphocytes[a]

| | Surface phenotype (%) | | | |
|---|---|---|---|---|
| *Patients* | *CD3* | *CD4* | *CD8* | *CD56* |
| 1 | 98 | 41 | 58 | 28 |
| 2 | 99 | 58 | 41 | 9 |
| 3 | 98 | 54 | 34 | 1 |
| 4 | 99 | 40 | 62 | 10 |
| 5 | 99 | 59 | 35 | 18 |
| 6 | 99 | 22 | 75 | nd |
| 7 | 99 | 16 | 83 | 27 |
| 8 | 98 | 75 | 18 | 21 |
| 9 | 99 | 10 | 85 | 13 |
| 10 | 89 | 21 | 61 | 15 |
| 11 | 93 | 29 | 66 | 4 |
| 12 | 99 | 36 | 63 | 28 |
| 13 | 99 | 36 | 63 | 28 |
| 14 | 95 | 26 | 66 | 7 |
| 15 | 94 | 53 | 41 | 18 |
| 16 | 91 | 34 | 68 | 10 |
| 17 | 90 | 48 | 38 | 21 |

[a]All lines express the 75 kDa TNF receptor; TNF-α mRNA was determined in lines 1, 2, 4, 7 and 10.

*Evaluation of mRNA transcripts for TNF-α*

To address the issue of whether IL-2-induced TIL proliferation is mediated by the release of TNF-α, we evaluated the ability of TILs to produce TNF-α and the role of anti-TNF-α antibody in IL-2-driven TIL proliferation. As shown in Figure 2, TIL lines obtained from five representative patients expressed a detectable message for TNF-α, thus indicating that this molecule is constitutively expressed in TILs cultured with IL-2. Detectable levels of TNF-α were also demonstrated in the supernatants obtained from all 13 TIL lines cultured in the presence of IL-2 (mean of all 13 lines $376.5 \pm 159.9\ pg\ ml^{-1}$). Owing to the low numbers of cells recovered, a Northern blot was not performed on freshly isolated TILs.

*Role of TNF-α and its receptors in IL-2-induced TIL growth*

Since TILs express the 75 kDa TNF-R and release TNF-α after *in vitro* culture with IL-2, in order to determine whether TNF-α and the 75 kDa TNF-R were involved in the proliferation induced by IL-2 TIL lines were cultured with different concentrations of IL-2 (10, 100, 1000 U $ml^{-1}$) in the presence or absence of anti-75 kDa TNF-R MAb and anti-TNF-α polyclonal antibody. All the TIL lines tested significantly proliferated in response to low and high concen-

**Figure 1** Immunofluorescent flow cytometric analysis of TNF receptors on two TIL lines (**a** and **b**) and on freshly isolated TILs from two patients (**c** and **d**) and assessment of PE–TNF-α binding (**e** and **f**) on two representative TIL lines. Relative cell number is indicated on the ordinate. The histograms of utr-1- and htr-9-stained cells were superimposed on the histogram of control IgG-stained cells (indicated by control). Marker was set up to include >95% of the control IgG-stained cells. **e** and **f**, Effects of pretreatment with control IgG, utr-1 and htr-9 MAbs on PE–TNF-α binding. TILs were pretreated with 20 μg $ml^{-1}$ utr-1 and 20 μg $ml^{-1}$ htr-9 before staining with PE–TNF-α. The staining with PE–TNF-α alone and with control IgG plus streptavidin (SA)–PE reagent alone is shown.

**Figure 2** Northern blot analysis of the expression of TNF-α in total RNA extracted from five TIL lines isolated from five patients with solid tumours (indicated as 1–5). Ten micrograms of total RNA were loaded per lane. The amount of loaded total RNA is shown following hybridisation for actin. The size of the messages is reported in the Materials and methods section.

**Figure 3** Effects of anti-TNF-α polyclonal antibody and anti-75 kDa TNF-R MAb on IL-2-driven proliferation of 13 TIL lines. TILs were cultured in the presence of different concentrations of IL-2 (10, 100 and 1000 U $ml^{-1}$) and in the presence of the above reported antibodies. The data are expressed as means ± s.d. percentage inhibition with respect to $^{3}$H-TdR incorporation by TILs cultured with IL-2 alone. ■, utr-1 MAb; ▨, anti-TNF-α Ab; □, control Ab.

trations of IL-2 (c.p.m. 16 500 ± 1208, 48 320 ± 6240, 56 430 ± 3480 at 10, 100 and 1000 U $ml^{-1}$ IL-2, respectively). These data are consistent with the phenotypic findings that these cells are equipped with a high-affinity IL-2R complex (Trentin *et al.*, 1994). When TIL lines were grown in the presence of different concentrations of IL-2 and anti-TNF-α antibodies (Figure 3) a discrete inhibitory effect was observed in the presence of this antibody, while control isotype antibody did not show any effects on IL-2-driven growth. The difference in percentage inhibition between anti-TNF-α MAb and control MAb was statistically significant ($P<0.05$). Proliferation assays following culture of TIL lines in

**Figure 4** Effects of anti-TNF-α polyclonal antibody and anti-75 kDa TNF-R MAb on IL-2-driven proliferation of freshly isolated TILs from a patient with lung cancer. TILs were cultured in the presence of different concentrations of IL-2 (10, 100 and 1000 U $ml^{-1}$) and in the presence of the above reported antibodies. The data are expressed as percentage inhibition of triplicate exeperiments with respect to the $^{3}$H-TdR incorporation by TILs cultured with IL-2 alone. ■, utr-1 MAb; ▨, anti-TNF-α Ab; □, control Ab.

the presence of different IL-2 concentrations and anti-75 kDa TNF-R MAb showed that utr-1 MAb was able to inhibit IL-2-driven TIL proliferation to different degrees (Figure 3), while control antibody did not ($P<0.05$). Repetitive experiments showed consistent results.

Freshly isolated TILs obtained from four patients were cultured in the same experimental conditions. The effect of anti-75 kDa TNF-R and anti-TNF-α antibodies in one representative subject is reported in Figure 4. Freshly isolated TILs did not proliferate in response to different concentrations of IL-2 as well as TIL lines (c.p.m. 6523 ± 308, 8305 ± 954, 21 308 ± 2154 at 10, 100 and 1000 U $ml^{-1}$ IL-2 respectively). When freshly isolated TILs were cultured in the presence of IL-2, both anti-75 kDa TNF-R MAb and polyclonal anti-TNF-α antibody displayed a decrease in the IL-2 mediated proliferation.

### Discussion

Our data demonstrate that TILs express the 75 kDa TNF receptor. Blocking this receptor resulted in an inhibition of TIL proliferation induced by IL-2. This suggests that TNF-R delivers a proliferative signal in TILs and that this effect is mediated by endogenously induced TNF-α following culture of TIL with IL-2, as demonstrated by Northern blot and ELISA analyses.

Although TILs have been reported to produce TNF-α (Belldegrun *et al.*, 1989; Wang *et al.*, 1989; Vaccarello *et al.*, 1990; Ioannides *et al.*, 1992) and this cytokine has been demonstrated to potentiate the lytic machinery of cytotoxic cells (Ostensen *et al.*, 1987; Ranges *et al.*, 1987; Espevik *et al.*, 1988; Blay *et al.*, 1989; Naume *et al.*, 1991), no information was available on the mechanisms used by TNF-α to deliver a proliferation signal to TILs. Our results confirm that TNF-α is constitutively expressed and released in TIL lines cultured with IL-2 and demonstrate that these cells bear the 75 kDa TNF receptor. Furthermore, our data clearly point to a specific role of TNF-α on TIL growth since anti-TNF-α and anti-TNF-R antibodies inhibit the IL-2-induced TIL proliferation. These observations suggest that

TILs might use their own secreted TNF-α in an autocrine/paracrine network. These observations are strengthened by the demonstration that freshly isolated TILs also bear TNF receptors (75 kDa) and that its blocking and/or adding anti-TNF-α antibody results in a decrease of IL-2-driven TIL proliferation.

To address the question of the specific surface structures involved in the transduction of the proliferative signal by endogenously produced TNF-α, TILs were evaluated for the presence of different TNF-Rs using MAbs that specifically recognise TNF receptors. Our results demonstrate that the 75 kDa TNF-R is expressed on the surface membrane of freshly isolated and cultured TILs, while the 55 kDa TNF-R was lacking on the same cells. In addition, the anti-75 kDa TNF-R antibody has been shown to affect the binding of PE-TNF-α. In view of the consideration that TNF-α induces a wide spectrum of activities, it is likely that some of these functions are independently mediated by one of the two receptors. In fact, the 55 kDa TNF-R has been observed to trigger cytotoxicity, the proliferation of fibroblasts and the synthesis of prostaglandin $E_2$, while the 75 kDa TNF-R delivers the signal for T-cell proliferation (Englemann *et al.*, 1990; Espevik *et al.*, 1990; Tartaglia *et al.*, 1991). Our observation that anti-75 kDa TNF-R antibody inhibits TIL proliferation suggests that this structure is involved in the *in vitro* TIL growth.

Our finding that TIL lines and freshly isolated TILs grow in the presence of IL-2 and express functional TNF receptors suggests that IL-2 might up-regulate the expression of this receptor, similar to what has been observed on cultured T cells obtained from peripheral blood (Ware *et al.*, 1991). The evidence that TNF-α up-regulates the expression of IL-2 receptors (Scheurich *et al.*, 1987; Chouaib *et al.*, 1988) indicates that the expression of these receptors (both IL-2 and TNF receptors) and the effect of these cytokines on T cells are likely to be closely related.

In as much as it has been extensively reported that TNF-α exhibits cytotoxic effects, the possible role of this cytokine in the control of tumour growth in neoplastic patients deserves comment. Results reported in this manuscript coupled with the observation that TILs release TNF-α following autologous tumour stimulation (Schwartzentruber *et al.*, 1991) highlight the role of this cytokine on the mechanism leading to the expansion and function of TILs. Moreover, the therapeutic efficacy of these cells on clinical grounds might be related to the role of TNF-α not only via a direct cytotoxic mechanism but also favouring the accumulation of relevant T-cell subsets at the site of tumour growth *in vivo*.

**Abbreviations**: TIL, tumour-infiltrating lymphocyte; TNF-α, tumour necrosis factor α; TNF-R, tumour necrosis factor receptor, PBL, peripheral blood lymphocyte; PE, phycoerythrin.

**Acknowledgements**

The authors wish to thank Biogen (Cambridge, MA, USA) for supplying recombinant IL-2; Dr M Brockhaus (Basle, Switzerland) for providing utr-1 and htr-9 MAbs; Dr G Trinchieri (Philadelphia, PA, USA) for providing B154.9 and B154.7 MAbs; Genentech (San Francisco, CA, USA) for kindly providing TNF-α cDNA; and Mr Martin Donach for his help in the preparation of the manuscript.

This study was supported by grants from the Italian Association for Cancer Research (AIRC, Milan), by the National Research Council (CNR, Rome), Project Clinical Applications of Oncological Research and by Progetto Finalizzato Regione Veneto. Drs L Trentin and R Zambello are recipients of a fellowship from the Ministero della Sanità, Istituto Superiore di Sanità (Rome); Dr P Bulian is a recipient of a fellowship from Associazione Italiana per la Ricerca sul Cancro (AIRC, Milan).

**References**

ANDREWS J, BERGER A AND WARE C. (1990). Characterization of the receptor for tumor necrosis factor (TNF) and lymphotoxin (LT) on human T lymphocytes. *J. Immunol.*, **144**, 2582-2591.

BALCH CM, RILEY LB, BAE YJ, SALMERON MA, PLATSOUCAS CD VON EA AND ITOH K. (1990). Patterns of human tumor-infiltrating lymphocytes in 120 human cancers. *Arch. Surg.*, **125**, 200-205.

BELLDEGRUN A, KASID A, UPPENKAMP M, TOPALIAN SL AND ROSENBERG SA. (1989). Human tumor infiltrating lymphocytes. Analysis of lymphokine mRNA expression and relevance to cancer immunotherapy. *J. Immunol.*, **142**, 4520-4526.

BLAY JY, BERTOGLIO J, FRADELIZI D AND CHOUAIB S. (1989). Functional interactions of IL2 and TNF in the differentiation of LGL into LAK effectors. *Int. J. Cancer*, **44**, 598-603.

BROCKHAUS M, SCHOENFELD H-J, SCHLAEGER E-J, HUNZIKER W, LESSLAUER W AND LOETSCHER H. (1990). Identification of two types of tumor necrosis factor receptors on human cell lines by monoclonal antibodies. *Proc. Natl Acad. Sci. USA*, **87**, 3127-3131.

CHOUAIB S, BERTOGLIO J, BLAY JY, MARCHIOL-FORUNIGAULT C AND FRADELIZI D. (1988). Generation of lymphokine activated killer cells: synergy between tumor necrosis factor and interleukin 2. *Proc. Natl Acad. Sci. USA*, **85**, 6875-6879.

CUTURI MC, MURPHY M, COSTA-GIOMI MP, WEINMAN R, PERUSSIA B AND TRINCHIERI G. (1987). Independent regulation of tumor necrosis factor production by human peripheral blood lymphocytes. *J. Exp. Med.*, **165**, 1581-1594.

ENGLEMANN H, NOVICK D AND WALLACH D. (1990). Two tumor necrosis factor-binding proteins purified from human urine. Evidence for immunological cross-reactivity with cell surface tumor necrosis factor receptors. *J. Biol. Chem.*, **265**, 1531-1537.

ESPEVIK T, FIGARI IS, RANGES GE AND PALLADINO MA. (1988). Transforming growth factor beta1 and recombinant tumor necrosis factor alpha reciprocally regulate the generation of lymphokine-activated killer cell activity. *J. Immunol.*, **140**, 2312-2318.

ESPEVIK T, BROCKHAUS M, LOETSCHER H, NONSTAD U AND SHALABY R. (1990). Characterization of binding and biological effects of monoclonal antibodies against a human tumor necrosis factor receptor. *J. Exp. Med.*, **171**, 415-426.

GRAY PW, BARRETT K, CHANTRY D, TURNER M AND FELDMANN M. (1990). Cloning of human tumor necrosis factor (TNF) receptor cDNA and expression of recombinant soluble TNF-binding protein. *Proc. Natl Acad. Sci. USA*, **87**, 7380-7384.

IMAMURA K, SPRIGGS D AND KUFE D. (1988). Expression of tumor necrosis factor receptors on human monocytes and internalization of receptor bound ligand. *J. Immunol.*, **139**, 2939-2942.

IOANNIDES CG, FISK B, TOMASOVIC B, PANDITA R, AGGARWAL BB AND FREEDMAN RS. (1992). Induction of interleukin-2 receptor by tumor necrosis factor-α on cultured ovarian tumor-associated lymphocytes. *Cancer Immunol. Immmunother.*, **35**, 83-91.

ITOH K, TILDEN AB AND BALCH CM. (1986). Interleukin-2 activation of cytotoxic T lymphocytes infiltrating into human metastatic melanoma. *Cancer Res.*, **46**, 3011-3017.

KAWAKAMI Y, ROSENBERG SA AND LOTZE MT. (1988). Interleukin 4 promotes the growth of tumor-infiltrating lymphocytes cytotoxic for human autologous melanoma. *J. Exp. Med.*, **168**, 2183-2191.

KIM TY, VON EA, FILACCIO MD, HAYAKAWA K, PARKINSON DR, BALCH CM AND ITOH K. (1990). Clonal analysis of lymphocytes from tumor, peripheral blood, and non tumor kidney in primary renal cell carcinoma. *Cancer Res.*, **50**, 5263-5268.

KNAPP W, DORKEN B, GILKS WR, RIEBER EP, SCHMIDT RE, STEIN H AND VON DEM BORNE AEGK. (1989). *Leukocyte Typing*, Vol. IV., Oxford University Press: Vienna.

KOHNO T, BREWER MT, BAKER SL, SCHWARTZ PE, KING MW, HALE KK, SQUIRES CH, THOMPSON RC AND VANNICE JL. (1990). A second tumor necrosis factor receptor gene product can shed a naturally occurring tumor necrosis factor inhibitor. *Proc. Natl Acad. Sci. USA*, **87**, 8331-8335.

KRADIN R, KURNICK JT, LAZARUS L, PREFFER F, DUBINETT S, PINTO C, GIFFORD J, DAVIDSON E, GROVE B, CALLAHAM R AND STRAUSS H. (1989). Tumor-infiltrating lymphocytes and interleukin-2 in the immunotherapy of patients with advanced cancer. *Lancet*, **ii**, 577-580.

LEWIS M, TARTAGLIA LA, LEE A, BENNETT GL, RICE GC, WONG GH, CHEN EY AND GOEDDEL DV. (1991). Cloning and expression of cDNAs for two distinct murine tumor necrosis factor receptors demonstrate one receptor is species specific. *Proc. Natl Acad. Sci. USA*, **88**, 2830–2834.

LOETSCHER H, PAN YC, LAHM HW, GENTZ R, BROCKHAUS M, TABUCHI H AND LESSLAUER W. (1990). Molecular cloning and expression of the human 55 kd tumor necrosis factor receptor. *Cell*, **61**, 351–359.

MUUL LM, SPIESS PJ, DIRECTOR EP AND ROSENBERG SA. (1987). Identification of specific cytolytic immune responses against autologous tumor in humans bearing malignant melanoma. *J. Immunol.*, **138**, 989–995.

NAUME S, SHALABY R, LESSLAUER W AND ESPEVIK T. (1991). Involvement of the 55- and 75-kDa tumor necrosis factor receptors in the generation of lymphokine-activated killer cell activity and proliferation of natural killer cells. *J. Immunol.*, **146**, 3045–3048.

NEDWIN GE, SVEDERSKY LP, BRINGMAN TS, PALLADINO MA AND GOEDDEL DV. (1985). Effect of interleukin-2, interferon-gamma, and mitogens on the production of tumor necrosis factor α and β. *J. Immunol.*, **135**, 2492–2498.

OSTENSEN ME, THIELE DL AND LIPSKY PL. (1987). Tumor necrosis factor alpha enhances cytolytic activity of human natural killer cells. *J. Immunol.*, **138**, 4185–4191.

PANDOLFI F, BOYLE LA, TRENTIN L, KURNICK JT, ISSELBACHER KJ AND GATTONI-CELLI S. (1991). Expression of HLA-A2 antigen in human melanoma cell lines and its role in T-cell recognition. *Cancer Res.*, **51**, 3164–3170.

PHILIP R AND EPSTEIN LB. (1986). Tumor necrosis factor as immunomodulator and mediator of monocyte cytotoxicity induced by itself, γ-interferon and interleukin 1. *Nature*, **323**, 86–88.

RANGES GE, FIGARI IS, ESPEVIK T AND PALLADINO MA. (1987). Inhibition of cytotoxic T cell development by transforming growth factor beta and reversal by recombinant tumor necrosis factor alpha. *J. Exp. Med.*, **166**, 991–997.

ROBINET R, BRANNELLEC D, TERMIJTELEN A, BLAY J, GAY F AND CHOUAIB S. (1990). Evidence for tumor necrosis factor-α involvement in the optimal induction of class I allospecific cytotoxic T cells. *J. Immunol.*, **144**, 4555–4561.

ROSENBERG SA, PACKARD BS, AEBERSOLD PM, SOLOMON D, TOPALIAN SL, TOY ST, SIMON P, LOTZE MT, YANG JC, SEIPP CA, SIMPSON C, CARTER C, BOCK S, SCHWARTZENTRUBER D, WEI JP AND WHITE DE. (1988). Immunotherapy of patients with metastatic melanoma using tumor infiltrating lymphocytes and interleukin-2: preliminary report. *N. Engl. J. Med.*, **319**, 1676–1680.

SCHALL T, LEWIS M, KOLLER KJ, LEE A, RICE GC, WONG GHW, GATANAGA T, GRANGER GA, LENTZ R, RAAB H, KOHR WJ AND GOEDDEL DV. (1990). Molecular cloning and expression of a receptor for human tumor necrosis factor. *Cell*, **61**, 361–370.

SCHEURICH P, THOMA B, UCER U AND PFIZENMAIER K. (1987). Immunoregulatory activity of recombinant human tumor necrosis factor (TNF)-α: induction of TNF receptors on human T cells and TNF-α-mediated enhancement of T cell responses. *J. Immunol.*, **138**, 1786–1790.

SCHWARTZENTRUBER DJ, TOPALIAN SL, MANCINI M AND ROSENBERG SA. (1991). Specific release of granulocyte-macrophage colony-stimulating factor, tumor necrosis factor-α, and IFN-γ by human tumor-infiltrating lymphocytes after autologous tumor stimulation. *J. Immunol.*, **146**, 3674–3681.

SHALABY MR, AGGARWAL BB, RINDERKNECHT E, SVEDERSKY LP, FINKLE BS AND PALLADINO MA. (1985). Activation of human polymorphonuclear neutrophil functions by interferon-γ and tumor necrosis factors. *J. Immunol.*, **135**, 2069–2075.

SHIMUZU Y, IWATSUKI S, HERBERMAN RB AND WHITESIDE TL. (1991). Effects of cytokines on in vitro growth of tumor-infiltrating lymphocytes obtained from human primary and metastatic liver tumors. *Cancer Immunol. Immunother.*, **32**, 280–288.

SMITH CA, DAVIS T, ANDERSO D, SOLAM L, BECKMANN MP, JERZY R, DOWER SK, COSMAN D AND GOODWIN RG. (1990). A receptor for tumor necrosis factor defines an unusual family of cellular and viral proteins. *Science*, **248**, 1019–1023.

TARTAGLIA LA, WEBER RF, FIGARI S, REYNOLDS C, PALLADINO MA AND GOEDDEL DV. (1991). The two different receptors for tumor necrosis factor mediate distinct cellular responses. *Proc. Natl Acad. Sci. USA*, **88**, 9292–9295.

TOPALIAN SL, SOLOMON D AND ROSENBERG SA. (1989). Tumor-specific cytolysis by lymphocytes infiltrating human melanomas. *J. Immunol.*, **142**, 3714–3725.

TRENTIN L, ZAMBELLO R, AGOSTINI C, AMBROSETTI A, CHISESI T, RAIMONDI R, BULIAN P, PIZZOLO G AND SEMENZATO G. (1990). Mechanisms accounting for the defective natural killer activity in patients with hairy cell leukemia. *Blood*, **75**, 1525–1530.

TRENTIN L, ZAMBELLO R, AGOSTINI C, SIVIERO F, ADAMI F, MARCOLONGO R, RAIMONDI R, CHISESI T, PIZZOLO G AND SEMENZATO G. (1993). Expression and functional role of tumor necrosis factor receptors on leukemic cells from patients with type B chronic lymphoproliferative disorders. *Blood*, **81**, 752–758.

TRENTIN L, ZAMBELLO R, BULIAN P, CERUTTI A, MILANI A, PIRONE E, NITTI D, AGOSTINI C AND SEMENZATO G. (1994). Functional role of IL-2 receptors on tumor infiltrating lymphocytes. *Br. J. Cancer*, **69**, 1046–1051.

VACCARELLO L, WANG YL AND WHITESIDE TL. (1990). Sustained outgrowth of autotumor-reactive T lymphocytes from solid tumors in the presence of tumor necrosis factor-alpha and interleukin-2. *Hum. Immunol.*, **28**, 286–292.

WANG YL, KANBOUR A, HERBERMAN RB AND WHITESIDE TL. (1989). Lymphocytes infiltrating human ovarian tumors: synergy between tumor necrosis factor-α and interleukin 2 in the generation of CD8+ effectors from tumor-infiltrating lymphocytes. *Cancer Res.*, **49**, 5979–5985.

WARE C, CROWE P, VANARSDALE T, ANDREWS J, GRAYSON M, JERZY R, SMITH C AND GOODWIN R. (1991). Tumor necrosis factor (TNF) receptor expression in T lymphocytes. Differential regulation of the type I TNF receptor during activation of resting and effector T cells. *J. Immunol.*, **147**, 4229–4238.

YAGITA M, ITOH K, TSUDO M, OWEN SCHAUB LB, PLATSOUCAS CD, BALCH CM AND GRIMM EA. (1989). Involvement of both Tac and non-Tac interleukin 2-binding peptides in the interleukin 2-dependent proliferation of tumor-infiltrating lymphocytes. *Cancer Res.*, **49**, 1151–1159.

ZAMBELLO R, TRENTIN L, BULIAN P, CASSATELLA M, RAIMONDI R, CHISESI T, AGOSTINI C AND SEMENZATO G. (1992). Role of tumor necrosis factor (TNF)-α and its specific 75 kd and 55 kd receptors in patients with lymphoproliferative disease of granular lymphocytes. *Blood*, **80**, 2030–2037.

ZAMBELLO R, TRENTIN L, PIZZOLO G, BULIAN P, MASCIARELLI M, FERUGLIO C, AGOSTINI C, RAIMONDI R, CHISESI T AND SEMENZATO G. (1990). Cell membrane expression and functional role of the p75 subunit of interleukin-2 receptor in a lymphoproliferative disease of granular lymphocytes. *Blood*, **76**, 2080–2085.